# T-2 mycotoxin: toxicological effects and decontamination strategies

**DOI:** 10.18632/oncotarget.15422

**Published:** 2017-02-16

**Authors:** Manish Adhikari, Bhawana Negi, Neha Kaushik, Anupriya Adhikari, Abdulaziz A. Al-Khedhairy, Nagendra Kumar Kaushik, Eun Ha Choi

**Affiliations:** ^1^ Department of Electrical and Biological Physics, Plasma Bioscience Research Center, Kwangwoon University, Seoul, Republic of Korea; ^2^ Department of Molecular Biology and Genetic Engineering, G B Pant University of Agriculture and Technology, Pantnagar, Uttarakhand, India; ^3^ Department of Life Science, Hanyang University, Seoul, Republic of Korea; ^4^ Department of Chemistry, Kanya Gurukul Campus, Gurukul Kangri Vishwavidyalaya, Haridwar, India; ^5^ Department of Zoology, College of Science, King Saud University, Riyadh, Saudi Arabia

**Keywords:** trichothecenes, oxidative damage, apoptosis, herbal antioxidant compounds, decontamination

## Abstract

Mycotoxins are highly diverse secondary metabolites produced in nature by a wide variety of fungus which causes food contamination, resulting in mycotoxicosis in animals and humans. In particular, trichothecenes mycotoxin produced by genus *fusarium* is agriculturally more important worldwide due to the potential health hazards they pose. It is mainly metabolized and eliminated after ingestion, yielding more than 20 metabolites with the hydroxy trichothecenes-2 toxin being the major metabolite. Trichothecene is hazardously intoxicating due to their additional potential to be topically absorbed, and their metabolites affect the gastrointestinal tract, skin, kidney, liver, and immune and hematopoietic progenitor cellular systems. Sensitivity to this type of toxin varying from dairy cattle to pigs, with the most sensitive endpoints being neural, reproductive, immunological and hematological effects. The mechanism of action mainly consists of the inhibition of protein synthesis and oxidative damage to cells followed by the disruption of nucleic acid synthesis and ensuing apoptosis. In this review, the possible hazards, historical significance, toxicokinetics, and the genotoxic and cytotoxic effects along with regulatory guidelines and recommendations pertaining to the trichothecene mycotoxin are discussed. Furthermore, various techniques utilized for toxin determination, pathophysiology, prophylaxis and treatment using herbal antioxidant compounds and regulatory guidelines and recommendations are reviewed. The prospects of the trichothecene as potential hazardous agents, decontamination strategies and future perspectives along with plausible therapeutic uses are comprehensively described.

## INTRODUCTION

**Figure 1:**
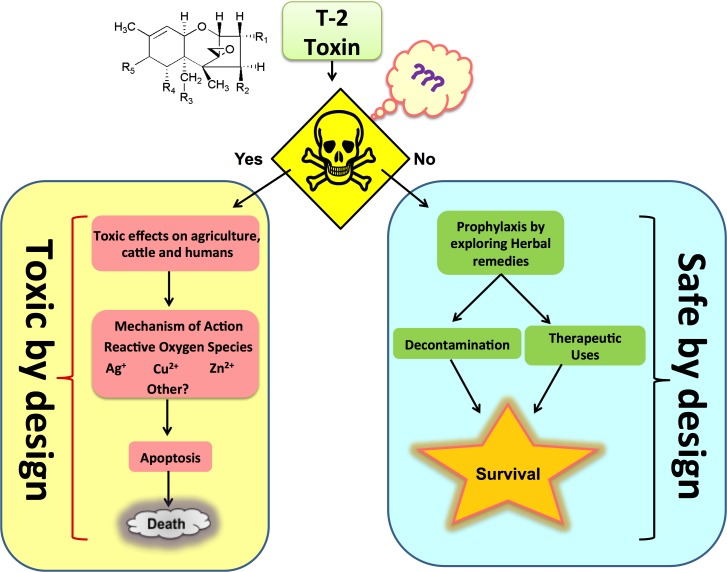
Schematic representation of T-2 toxin by its toxic and safe design

Mycotoxins are a group of chemically assorted compounds originating from the secondary metabolism of molds (filamentous fungi) that causes many diseases. The far, more than 300 mycotoxins have been found to induce toxicological effects in mammals only [1]. It is estimated that approximately 25% of the world’s agricultural commodities are contaminated to some extend with mycotoxins [2, 3]. Such studies revealing necessarily high occurrences and concentrations of mycotoxins suggest that mycotoxins are a constant concern. The synthesis of mycotoxins very closely resembles those processes that utilize primary metabolic pathways, such as amino acid and fatty acid metabolism. Toxin production and the degree of contamination of feed and food commodities are regulated by environmental factors such as the substrate composition and the texture, temperature and humidity. The genera of mycotoxin-producing fungi are *Aspergillus, Fusarium, Penicillium, Alternaria, Phomopsis, Emericella, Cephalosporium, Myrothecium, Trichoderma, Trichothecium, Neopetromyces, Byssochlamys, Neotyphodium* and *Claviceps*. The adverse effect of fungal products have instigated mass poisoning in both man and farm animals in many countries [1]. The main mycotoxins, the fungi producing them, and associated commodities are presented in Table 1. T-2 toxins are agriculturally among the most important mycotoxins that present a potential hazard to health worldwide. These compounds are derivatives of a ring system referred to as trichothecenes [4]. T-2 toxins belong to a large family of chemically related toxins produced by fungi in taxonomical genera such as *Fusarium, Myrothecium* and *Stachybotrys*. There are more than 20 naturally occurring compounds produced by the *Fusarium* species with similar structures, including diacetoxyscirpenol, nivalenol, deoxynivalenol, the T-2 toxins, HT-2 toxin and fusaron X [5]. In this review, we discuss the toxic effects of T-2 toxins on agriculture, livestock and humans and also simultaneously report safety information regarding survival against the harmfulness of these toxins (Figure 1).

**Table 1:** Mycotoxins and its related fungus with contaminating foods

Mycotoxin	Food Products	Related fungi
Aflatoxins	Cereals, oil seeds, spices, dry fruits, other nuts and corn	*Aspergillus parasiticus, A. flavus*
Fumonisins	Mainly in cereals and corns	*Fusarium moniliforme, F. culmorum, F. avenaceum, F. proliferatum, F. verticillioides, F. nivale, Gibberella fujikuroi*
Ochratoxin	Cereals, legumes, coffee beans	*Aspergillus ochraceus, Penicillium verrucosum/viridicatum*
Patulin	Grapes, apples, other fruits	*Aspergillus clavatus, A. giganteus, Penicillium expansum, Botrytis, P. roquefortii, P. claviforme, P. griseofulvum,* other *Penicillium* and *Aspergillus sp.*
Trichothecenes (T-2/toxins)	Wheat, corn	*Fusarium moniliforme, F. equiseti, F. culmorum, F. solani, F. avenaceum, F. roseum, F. nivale Fusarium tricinctum, F. poae, F. sporotrichiella, F. graminearum* and other fungal species

## CHEMICAL STRUCTURE OF T-2 TOXINS

**Figure 2:**
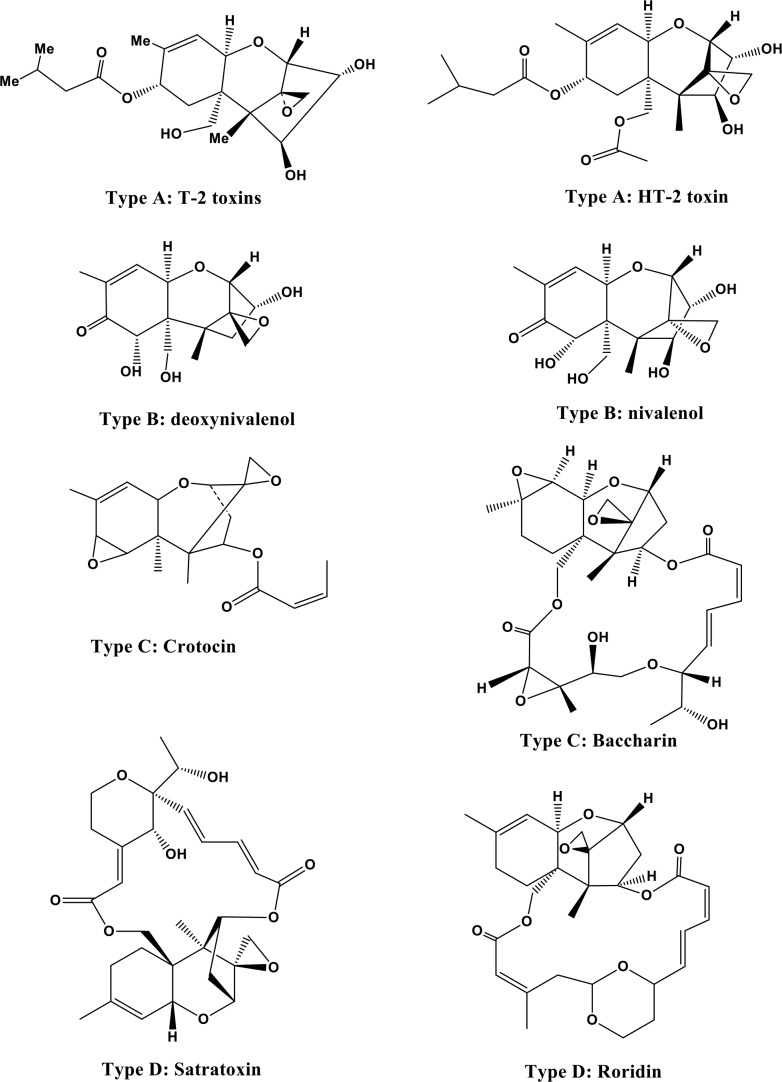
Structures of T-2 and HT-2 toxins (type A) and other trichothecenes (types B, C, and D)

Trichothecenes have a tetracyclic sesquiterpenoid 12,13-epoxytrichothec-9-ene ring in common (Figure 2), and the 12,13-epoxy ring which is responsible for the toxicological activity [6]. Their chemical structure is characterized by hydroxyl (OH) group at the C-3 position, acetyloxy (-OCOCH_0_) groups at the C-4 and C-15 positions, hydrogen at the C-7 position, and an ester-linked isovaleryl [OCOCH2CH(CH_3_)_2_] group at the C-8 position [7].

**Figure 3:**
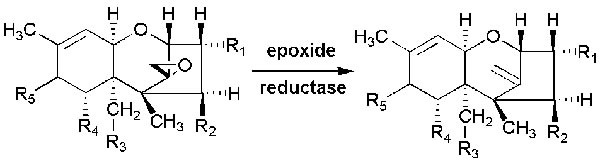
Microbial transformation of trichothecenes into their de-epoxylated forms

On the basis of characterized functional groups, trichothecenes can be classified into four groups. Type A trichothecenes are mainly represented by T-2 toxins (henceforth T-2 or the T-2 toxin) and the HT-2 toxin (HT-2) and do not contain a carbonyl group at the C-8 position (Figure 2). In type B trichothecenes, a carbonyl group is present at the C-8 position. The main representatives of type B trichothecenes are deoxynivalenol and nivalenol (Figure 2). Trichothecenes of type C (e.g., crotocin and baccharin) have a second epoxy ring between C-7 and C-8 or between C-9 and C-10. Trichothecenes of type D, such as satratoxin and roridin, contain a macrocyclic ring between C-4 and C-15. The T-2 toxin has ability to undergoes microbial transformation and converts into its deepoxylated form [8] (Figure 3) in the intestine which is very important in toxic-reducing pathway.

## CHEMICAL SYNTHESIS AND PROPERTIES OF THE T-2 TOXIN

T-2 is nonvolatile and resilient to degradation in diverse environments, such as those with different light and temperature levels, but it is deactivated easily by strongly acidic or alkaline conditions. The synthesis of T-2 starts from trichodiene, isolated from *T. roseum* [9] and *F. culmorum* [10,11]. T-2 toxin prepared through the sequence of oxygenations, cyclizations, isomerizations and esterification of trichodiene in several laboratories in the United States, Canada and England. Chemically, the T-2 toxin is insoluble in water but soluble in acetone, ethyl acetate, chloroform, ethanol, methanol and propylene glycol, though it is stable in diverse environmental conditions, even when autoclaved. T-2 may also be decreased by the presence of coexisting bacteria or fungi that can detoxify it by altering its chemical structure [12, 13, 14]. In order to achieve inactivation, it should be heated to 900°F for 10 min or 500°F for 30 min [15].

## HISTORICAL SIGNIFICANCE

In 1940, Soviet scientists coined the term stachybotryotoxicosis to describe an acute syndrome consisting of a sore throat, bloody nasal discharge, dyspnea, cough, and fever resulting from inhalation of the *Stachybotrys* mycotoxin. The potential use of the T-2 mycotoxin as a biological weapon was realized during World War II in Orenburg, Russia when civilians consumed wheat that was unintentionally contaminated with *Fusarium* fungi. The victims developed a protracted lethal illness with a disease pattern similar to that of alimentary toxic aleukia (ATA). Twenty years after of this incident, the trichothecene mycotoxin was discovered and the T-2 toxin was isolated [16].

## ECOLOGICAL PREVALENCE AND FACTORS STIMULATING TOXIN PRODUCTION

T-2 and HT-2 toxins are predominantly found in grains, such as wheat, maize, barley, rice, soybeans and particularly in oats and products thereof [17]. The fungal propagation and production of mycotoxins is enhanced in developing countries around the world due to tropical conditions like high temperatures and moisture levels, monsoons, unseasonal rains during harvests and flash floods. It has been reported that cereals grown in the humid subtropical climate regions of China, Thailand, Vietnam and South Korea can show evidence of the prevalent growth of *Fusarium sp*. The production of mycotoxins is enhanced by factors such as the humidity of the substrate (10 to 20%), the relative humidity (≥ 70%), the temperature (0 to 50°C, depending on the fungus species) and the availability of oxygen [18]. Researchers have noted that crops in tropical and subtropical areas are more susceptible to mycotoxin contamination as compared to those in temperate zones due to the presence of high humidity and temperatures in tropical areas, which provide optimal conditions for toxin formation [19]. The major factors that are important in the production of mycotoxins during the pre-harvest and post-harvest handling of agricultural products [20] are as follows:

- Intrinsic factors consisting of the moisture content, water activity, substrate type, plant type and nutrient composition;
- Extrinsic factors such as the climate, temperature, and oxygen level;
- Processing factors including drying, blending, the addition of preservatives, and the handling of grains;
- Implicit factors such as mainly insect interactions, fungal strains, and the microbiological ecosystem.

## ROUTES OF EXPOSURE AND TRANSMISSION

The trichothecene mycotoxins are readily absorbed by various modes, including the topical, oral, and inhalational routes. As a dermal irritant and blistering agent, it is alleged to be 400 times more intoxicating than sulfur mustard. Respiratory ingestion of the toxin indicates its activity being comparable to that of mustard or lewisite [21]. The T-2 mycotoxin is distinctive in that systemic toxicity can result from any route of exposure, *i.e*., dermal, oral, or respiratory [16]. Some insects such as *Sitobion avenae* (aphid) help in transmitting the *Fusarium langsethiae* inoculum to infect humans [22]. Transmission can occur by direct exposure of contaminated objects and surfaces that have not been appropriately decontaminated.

## TOXICITY OF THE T-2 TOXIN

The trichothecene family boasts of a wide range of toxins, and T-2 is one of the earliest investigated and amongst the most toxic members of the family as compared to other mycotoxins (Table 2). The toxicity and deleterious effects of T-2 vary on the basis of numerous factors, such as the route of administration; the time and amount of exposure; the dosage administered; and the age, sex and overall health of the animal along with presence of any other mycotoxin [23]. Intoxication often occurs after feeding on feed made from grain, hay and straw, wintering in the open and becoming contaminated with *F. sporotrichiella* and *F. poae*. Poisoning in humans is known as alimentary toxic aleukia. The toxins produced by these species (T-2 and Diacetoxyscirpenol) have a local irritant effect and cause serous hemorrhagic inflammation; necrosis and ulceration in the digestive tract; and dystrophy in liver, kidney, heart, brain and peripheral ganglia of the vegetative nervous system. Damage is seen in the blood vessel walls, and hemorrhagic diathesis is provoked [24]. The T-2 toxin also helps in inducing cytotoxicity and damage in mouse immature Leydig cells (TM3) [25]. The metabolic pathways are also altered in different organs, such as the spleen, thymus, stomach and liver in Wistar rats after T-2 toxin exposure [26]. The increased elevation of glutathione disulfide and 3-hydroxybutyrate suggest that the T-2 toxin promotes an anti-oxidative response in organ systems and helps with free radical generation. In addition, the depletion of urinary l-methylniconate and 1-methylnicotinamide can occur during cysteine biosynthesis (Figure 4). The T-2 toxin caused reductions of succinate and citrate in urine and a reduced level of fumarate in the liver, accompanied by an increase in NAD at high levels in rats exposed to T-2, suggesting that T-2 lowers the rate of the tricarboxylic acid (TCA) cycle. The results in Figure 4 suggest that the T-2 toxin induces oxidative stress in rats exposed to the T-2 toxin.

**Table 2:** Relative toxicity of different mycotoxins on different livestock species

# = Slight toxicity ## = Adequate toxicity ### = High toxicity			
Toxin	Poultry	Ruminants	Swine
Aflatoxins	###	#	##
T-2 toxins	##	###	###
Ochratoxin	###	#	#
Zearalenone	#	##	###
Fumonisin	#	#	###
Deoxynivalenol	#	##	##

**Figure 4:**
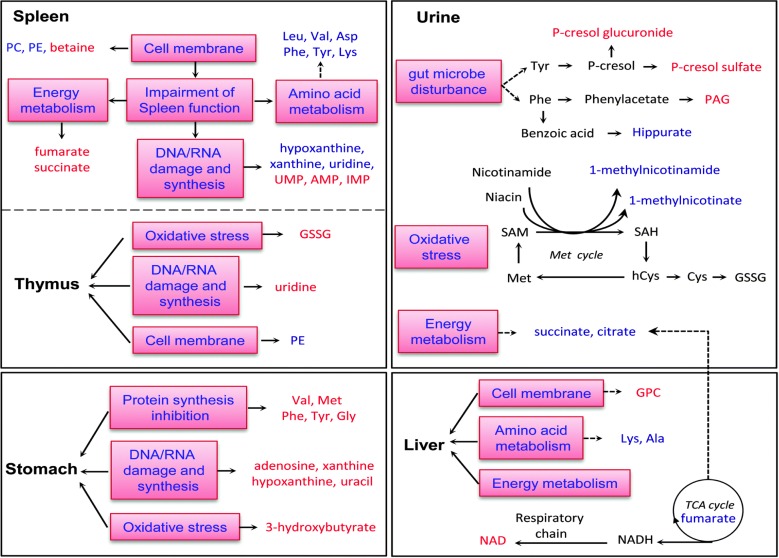
Diagrammatic representation of altered metabolic pathways in different organs of Wistar rats followed by T-2 toxin treatment Metabolites shown in red or blue denote a significant increase or decrease in T-2 toxin treated rats with respect to control rats. Metabolites shown in black denote no marked change. (Reproduced from Wan Q et al. 2015 Mol. Biosyst. with permission of The Royal Society of Chemistry).

### Acute toxicological effects

As described above, the effects of the toxin can be revealed through multifarious means of exposure. It is the only toxin in this family of toxins that can be absorbed directly through the skin. The chiefly illustrative symptoms of T-2 toxicity are emesis, vomiting, skin blistering, loss of appetite and weight loss. Table 3 depicts the probable exposure routes and LD_50_ values of the toxin in various experimental models.

**Table 3:** LD_50_ values of T-2 toxin in different animals with different administration pathways

Species	Mode of administration	LD50 (mg/kg bw)	References
Mice	Oral	10	Ueno 1984
Mice	Intraperitoneal	5.2	Ueno 1984
Mice	Subcutaneous	2.1	Ueno 1984
Mice	Intravenous	4.2	Ueno 1984
Rats	Intraperitoneal	1.5	Creasia et al. 1990
Rats	Subcutaneous	1.0	Bergmann et al. 1985
Rats	Intramuscular	0.85	Chan et al. 1984
Rats	Intravenous	0.9	Fairhurst et al. 1987
Rats	Inhalation	0.05	Creasia et al. 1990
Guinea Pigs	Intraperitoneal	1.2	Creasia et al. 1990
Guinea Pigs	Intravenous	1-2	Fairhust et al. 1987
Guinea Pigs	Inhalation	0.4	Creasia et al. 1990
Rabbits	Intramuscular	1.1	Chan et al. 1984
7-days-old broilers	Oral	4	Hoerr et al. 1981
Pigs	Intravenous	1.21	Weaver et al. 1978

Studies have been conducted to assess acute toxicity levels in various experimental models, including mice, guinea pig, pigeons and rats which have been administered the T-2 toxin using different exposure routes, viz. subcutaneous, intratracheal, intravenous, intraperitoneal, and intragastric [27]. It was observed that rats administered the T-2 toxin exhibited elevated brain concentrations of tryptophan and serotonin, which led to an upsurge in dopamine and a consequential decline in 3,4-dihydroxyphenylacetic acid levels [28]. Additionally, it was observed that as the concentration of dopamine increased, epinephrine levels declined in adrenal glands. This cascade reaction indicates that T-2 induces the elevation of indoleamine levels in the brain, causing animals to show feed refusal behavior [29]. The T-2 mycotoxin alters the development of mouse blastocysts, reduces the number of blastomeres, and increases chromatin damage [30]. However, T-2 along with the HT-2 mycotoxin in laboratory animals fed a commercial feed were not detected if the levels are between 250 and 2000μg/kg body weight [31]. An analysis of acute toxicity studies in a rabbit model displayed pathological and histopathological changes in the GI tract, bone marrow and lymphocytes. In contrast, subacute toxicity demonstrated catarrhal gastritis involving inflammation of the stomach lining, hypertrophy and emaciation of adrenal cortex [32].

### Chronic toxicological effects

Chronic effects of exposure to the T-2 toxin were characterized in female rats as an upsurge in tyrosine and serotonin levels in the cerebellar region. In addition, an elevation in cortical tryptophan levels was also observed, indicating variation in the T-2 toxin mode of action in terms of chronic effects, in contrast to acute administration behavior [33]. It was observed that while an acute systemic T-2 treatment elevated tryptophan levels, a decline in serotonin levels was noted simultaneously in the cerebellar and brainstem regions [33]. Numerous other symptoms are also associated with chronic toxicity, including emaciation, necrosis in the lymphoid tissue and subacute catarrhal gastritis in rabbits [34]. Consumption of feed contaminated with T-2 has shown to reduce weight gain and egg production as well as the egg hatching ability in chickens. In addition, substantial declines in serum cholesterol and total protein levels as well as elevations in lactate dehydrogenase and uric acid levels in serum samples were also reported in various studies [35, 36, 37, 38]. Another phenotypic alteration stemming from T-2 toxicity includes feather alteration in chickens [39]. The toxic effect of T-2 administration was also evident in a study of white Pekin ducks, which demonstrated a marked decline in their weight gain ability with increasing T-2 toxin dose concentrations [40]. The study also revealed that the blastogenic response of lymphocytes against specific and nonspecific mitogens was also strikingly impaired [41]. Hence, it is evident that T-2 had toxic effects in animals, such as weight loss, decreased blood cell and leukocyte counts, decreased plasma glucose levels, and certain pathological effects and lining changes in the liver and stomach. Additionally, T-2 is linked to an increased infection rate, DNA damage and the induction of apoptosis [29, 42, 43, 44, 45].

### Effects on dairy cattle

Ruminants are known to be relatively resistant to the T-2 toxin in comparison to monogastric animals. The primary cause of this phenomenon is principally considered to be the de-epoxidation and de-acetylation activity in the rumen for the protection of cows against T-2-induced toxicity [46]. After absorption in stomach, the toxic symptoms are dominated by the cytotoxic action on the bone marrow. T-2 toxin exposure has been associated with feed refusal, production losses, gastroenteritis lesions, intestinal hemorrhages and death in dairy cattle. The lesions in the oral cavity are weaker - only hyperaemia and edema of the oral mucosa are usually seen, whereas hyperaemia and hemorrhaging of the mucosa of the abomasus are often present. Tremors and paralysis of the hind limbs are often seen, but the haemorrhagic diathesis is less pronounced than in cases involving other species [47]. It has also been assumed to exert reduced immune responses in calves. Various studies have reported that the toxic effects of T-2 toxin result in bloody feces, enteritis, abomasal and ruminal ulcers, and death. Symptoms such as decreased milk production and the absence of estrus cycles in cows have also been attributed to exposure of T-2. Serum immunoglobulins, complement proteins, and white blood cell and neutrophil counts were demonstrated to be lower in calves exposed to the T-2 toxin [48]. Experimental evidence shows that lambs fed the T-2 toxin develop symptoms of focal hyperemia and dermatitis at the mucocutaneous junction of the commissure of the lips, along with diarrhea, leukopenia, lymphopenia and lymphoid depletion of mesenteric lymph nodes and the spleen [46].

### Effect on poultry

In poultry, the T-2 toxin has been the causative agent for mouth and intestinal lesions in addition to the impairment of immune responses, destruction of the hematopoietic system, declining egg production, the thinning of egg shells, refusal of feed, weight loss and altered feather patterns, abnormal positioning of the wings, hysteroid seizures or an impaired righting reflex [49, 50]. It has been reported that poultry are relatively less susceptible to trichothecenes than pigs. The serous-haemorrhagic necrotic-ulcerative inflammation of the digestive tract with thickening of the mucosa, a lurching gait/step, and refusal of food due to oral lesions are the main symptoms observed. It was observed that acute intoxication of broiler chickens exhibits consequences consisting of internal hemorrhaging, mouth and skin lesions (necrohemorrhagic dermatitis), impaired feather quality and neural disturbances [51]. Significantly reduced levels of haemoglobin and the packed cell volume in intoxicated broiler chicks have been observed at low doses as well. Decreases in serum total protein and cholesterol levels and increases in serum uric acid and lactate dehydrogenase levels were also exhibited upon T-2 exposure, hence conclusively indicating that toxic effects of T-2 are evident on performance, biochemical and immunological parameters even at very low levels in broiler chicks [38, 52]. A patho-histological survey usually reveals fatty changes/dystrophy and strong granular degeneration in the liver, kidneys and rarely in the heart. Necrosis in the digestive tract is superficial. In chronic stages, interstitial nephritis, kidney sclerosis and glomerulonephritis are seen, and the necroses in the stomach and intestines become profound [47].

### Effects on pigs

Along with the serous-haemorrhagic necrotic-ulcerative inflammation of the digestive tract, some necroses are established on the snout, lips and tongue, edema and mucous coatings of the mucosa of the stomach, swelling in the region of the head, especially around the eyelids and larynx, and rarely, paresis or paralysis are seen [47].

Toxic effects of the T-2 toxin are usually manifested in the form of alimentary toxic aleukia (ATA) in pigs. The symptoms include vomiting, diarrhea, leukopenia, hemorrhage, shock and death. Acute toxicological effects are also characterized by multiple hemorrhages of the serosa of the liver and along the intestinal tract, stomach and esophagus (at necropsy). The presence of blood was reported in intestines and in the abdominal cavity, and a cream-colored paste was noted on the lining of the esophagus and the ileum [18]. Low dosage chronic exposure resulted in growth retardation, weight gain suppression and feed refusal [51]. Variation in exposure levels to the toxin also exerts diverse effects on the immune system of animals. For instance, low concentrations induce pro-inflammatory gene expression at the mRNA and protein levels, whereas high concentrations have been observed to promote leukocyte apoptosis [51].

Experimental evidence indicates that exposure to the T-2 toxin results in lesions in the stomach associated with congestion, hemorrhages and the presence of necrotic cells in the isthmus and neck regions. Symptoms of submucosal edema and necrotic crypt epithelial cells were observed in the duodenum, jejunum, ileum, cecum and colon, with the most severe lesions being in the colon. The same study indicated higher lymphocyte depletion and necrosis levels in the lymph node cortex region as compared to the paracortex. Apoptotic bodies were observed in intestinal crypt cells, lymphoid cells from the lamina propria, and ileal Peyer’s patches, indicating apoptosis as the major mechanism of action involved in intestinal lesions due to T-2-induced toxicity [53]. T-2 contamination of feedstock has been reported to result in decreased red blood cell counts, and decreases in the MCV and hemoglobin levels of red blood cells. A significant reduction in the number of T lymphocytes was also observed. Feed contamination also has an inhibitory effect on the ovaries, with histological degeneration and accompanying atrophy [18]. HT-2 toxin exposure causes oxidative stress which induces apoptosis/autophagy in porcine oocytes [54]. Additionally, toxin exposure can have reproductive and teratogenic effects but exerts no carcinogenic effect [51].

### Effects on horses

In addition to the described symptoms of ulceration and necrosis of the mouth mucosis, gray-white coatings on the tongue and palate, spasms and tremors of some muscles, and occasional paresis of the hind limbs have been seen [47]. Despite studies of the contamination of cereals and animal feed with trichothecenes [55, 56], little is known about the characteristics of equine exposure to these mycotoxins [57, 58, 59]. The long-term effects of the administration of T-2 toxins in mares were evaluated with a daily oral dosage of 7mg of the pure T-2 toxin for 32-40 days. No effects on fertilization or ovarian activity were detected in mares, though oral lesions were detected in some cases [60].

## MECHANISM OF ACTION

The T-2 toxin have thiol group makes it a potent protein and DNA synthesis inhibitor [23]. It also reduces lymphocyte proliferation, alters the membrane function, impairs the production of antibodies and alters the development of dendritic cells [61]. The T-2 toxin causes apoptosis in various cell types *in vitro*, such as human liver cells, HL-60 cells, Jurkat cells, U937 cells, and Vero cells. Deleterious effects are also manifested in a mice model, exhibiting apoptosis in various tissues and organs including the skin, kidney, brain and bone marrow [16]. The appearance of these manifestations constitutively is mainly due to oxidative damage to cells that targets biomolecules such as lipids, proteins and nucleic acids. The main ROS involved in the oxidation of proteins, lipids and DNA appear to be hydrogen peroxide, hydroxyl radical and superoxide molecules. The mitochondrial complex I and CYP450 have also been reported to be involved in mycotoxin-induced ROS generation [62]. Furthermore, the T-2 toxin can decrease the function of the innate immune system [63].

**Figure 5:**
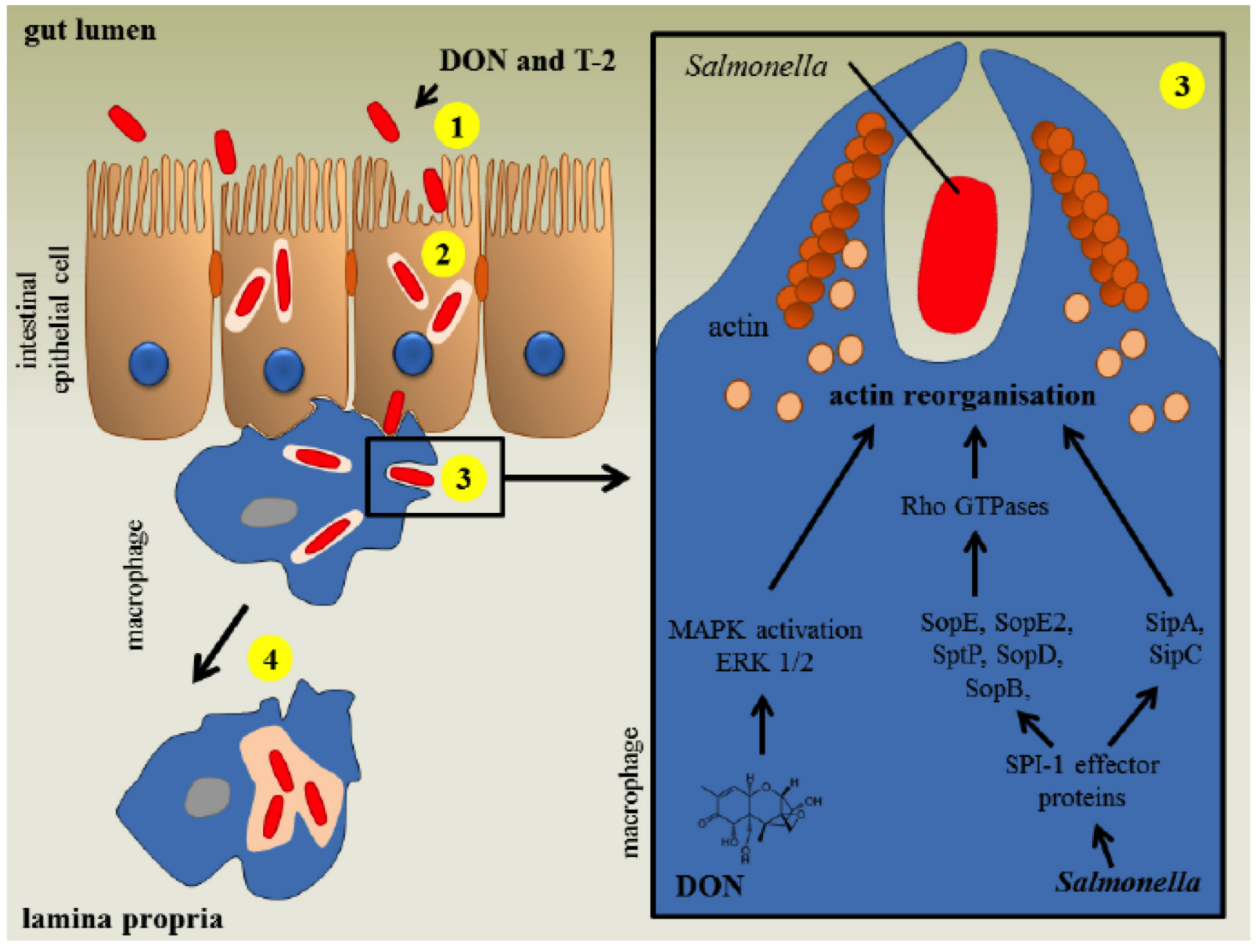
The impact of T-2 mycotoxins on the human intestinal gut region against infection by salmonella (Reproduced from Antonissen et al., 2014, with the permission of the Toxin Journal).

Typically, the T-2 toxin is hypothesized to bind and inactivate peptidyl-transferase activity at the transcription site [64], resulting in the inhibition of protein synthesis [16]. The most prominent molecular target of trichothecenes includes the 60S ribosomal unit, where it prevents polypeptide chain initiation [51]. This inhibitory effect is most prominent in actively proliferating cells, for instance those of the skin and gastrointestinal tract, the bone marrow and thyroid, and erythroid cells [65]. Moreover, the T-2 toxin is believed to disrupt DNA polymerases, terminal deoxynucleotidyl transferase, monoamine oxidase and several other proteins involved in the coagulation pathway [66]. This toxin has been shown to decrease the colonization capacity of *Salmonella* in pigs [67]. The T-2 toxin enhances the uptake of *Salmonella* in macrophages via the activation of the mitogen-activated protein kinase (MAPK) extracellular signal-regulated kinase (ERK1/2) pathway, which induces actin reorganization and membrane ruffles (Figure 5).

The mechanism of action of trichothecenes involves the interaction of the toxin with subcellular structures, resulting in the disruption of the mitochondrial morphology, rough endoplasmic reticulum and other membranes [16]. They act upon and hinder the activity of metabolically critical enzymes such as succinic dehydrogenase, consecutively impeding cellular energetics by decreasing the oxidation of succinate, malate and pyruvate molecules and additionally inhibiting protein synthesis in mitochondria [23]. Moreover, the ability to cross the placenta and damage mouse fetuses via the acceleration of cell death by apoptosis in the immune system and other tissues has also been documented in the trichothecenes family [68]. However, oxidative stress due to the T-2 toxin in rat hepatocytes can be reduced using l-carnitine [69]. It also regulates steroid hormone secretion through the cAMP-PKA pathway in rat ovarian granulosa cells [70].

## GENOTOXIC AND CYTOTOXIC EFFECTS

The T-2 toxin is known to impact the synthesis of biomolecules such as DNA, RNA and proteins, thus inhibiting cellular functions such as the cell cycle and resulting in apoptosis [42] [71]. Due to its structural distinctiveness along with the HT-2 toxin, unlike other trichothecenes, it impedes protein synthesis by inhibiting the polypeptide chain initiation process. Toxicity of the T-2 toxin on living beings has been stated in terms of its deleterious effects on lymphoid cells and its diminishing effects on the immune system [72].

The toxin primarily exerts effects similar to those of a radiation injury by negatively impacting protein levels and RNA and DNA synthesis processes [72, 61]. Studies involving Chinese hamster V79 cells have indicated that it induces micronuclei formation, gene mutations and sister chromatid exchanges and also results in hindering intercellular cross-talk.

### Apoptosis

**Figure 6:**
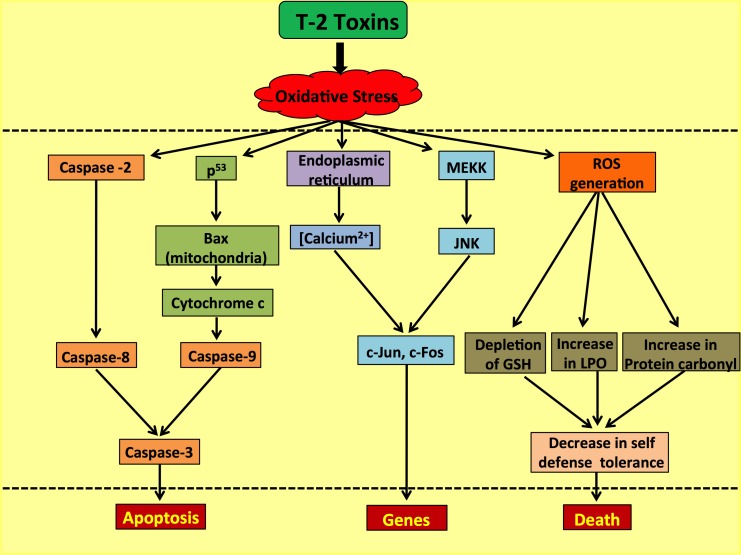
Role of the T-2 toxin in causing ROS-mediated caspase-dependent and independent apoptosis in human cells

The ERK1/2 pathway and the JNK/p38 MAP kinase pathway activated by stress responses play a pivotal role in determining the prospects of cell survival or the undergoing of apoptosis. Hence, maintenance of homeostasis amongst these pathways is essential for cellular survival. However, the T-2 toxin and its metabolites have been observed to induce apoptosis by the activation of c-Jun N-terminal kinase 1 (JNK1) and/or p38MAPK (SAPK2), also triggering the stimulation of MAP kinases involved in regulating cellular proliferation of, for instance, ERK1/2 [73]. Another group of scientists found an additional pathway in which the T-2 toxin generates pro-apoptotic conditions in the cellular milieu by initiating a cascade reaction involving Fas up-regulation on chondrocyte surfaces, followed by the up-regulation of p53 proteins which in turn increases the Bax/Bcl-2 and Bax/Bcl-xL ratios, simultaneously activating the caspase-3-dependent apoptotic pathway [74]. T-2 toxin along with satratoxin G induce DNA damage that involves activation of ATM pathway which alter checkpoint kinase Chk2 [75] and leads to apoptosis. A separate study has established that deleterious effects of the T-2 toxin are intermediated by ROS generation which contributes to DNA damage and enhancement of the p53 protein expression in HeLa cells (Figure 6).

This p53 protein activation causes an alteration of the Bax/Bcl-2 ratio, leading to caspase-dependent apoptosis mediated by a mitochondrial Cyt-c release [76]. In addition, Figure 6 demonstrates that HeLa cells exposed to the toxin are damaged by the autonomous activation of the AIF pathway independent of the caspase cascade, subsequently causing DNA fragmentation, apoptosis and eventually cell death [76].

### Inhibition of T-2-toxin-induced apoptosis

The T-2 toxin has been documented to induce apoptosis in human chondrocytes via Bcl-2 and Bax proteins. Additionally, it is well known that the Bax/Bcl-2 ratio pathway plays a pivotal role in determining cellular susceptibility to undergo apoptosis. It has been found that selenium can partly block chondrocyte apoptosis induced by the T-2 toxin by reducing the Bax/Bcl-2 ratio [77]. Another independent study ascertained that nano-Se-chondroitin sulfate can inhibit the T-2-toxin-induced apoptosis of cultured chondrocytes derived from Kashin-Beck disease (KBD) patients *in vitro* [78]. A further assessment of T-2-toxin-induced chondrocyte apoptosis elucidated that increased levels of ATF2, JNK and p38 mRNAs and related protein expression levels play a vital role in apoptosis induction. It was also noted that the JNK and p38 pathways were involved in the apoptosis induced by the T-2 toxin in chondrocytes. It was revealed that selenium chondroitin sulfate nanoparticles (SeCS) can partly block apoptosis by decreasing the expressions of ATF2, JNK and p38 mRNAs and the p-JNK, p-38, ATF2 and p-ATF2 proteins [79].

## DETERMINATION OF THE T-2 TOXIN: METHODS AND TECHNIQUES

Several methods for the determination of the T-2 toxin based on traditional chromatographic, immunoassay, or mass spectroscopy (MS) techniques have been studied thus far [68]. Gas-liquid chromatography (GLC) and high-pressure liquid chromatography (HPLC) with MS may be used to assess the presence of T-2 and related trichothecene mycotoxins in plasma and urine samples [80]. 50-75% of the ingested toxin and metabolites are eliminated in the urine and feces within 24 hours. Early post-exposure (0-24 hours) nasal or throat swabs and induced respiratory secretions can be used for detection by HPLC/GLC/MS and immunoassay methods. During the last decade, liquid chromatography with mass spectrometry has become the most recurrently used technique for the determination of T-2 and HT-2 toxins, often within a multi-analyte approach [17]. Hence, the estimated daily intakes were found to be 2.56, 3.22, 2.53, 0.03, 0.01 and 2.45 ng (kg bw), for brown rice, barley, mixed grains, corn, wheat and wheat flour, respectively (Table 4) [81].

**Table 4:** Estimated daily intakelevels and total T-2 and HT-2 toxins present in cereals and cereal-based products by assuming a body weight of 55 kg

Samples	Consumption (g/day)	Total Toxins present (μg/kg)	Estimated daily intake [ng/kg bw/day]
Brown Rice	2.92	48.3	2.56
Barley	6.71	26.4	3.22
Mixed Grains	4.6	30.2	2.53
Corn	0.03	63.0	0.03
Wheat	0.02	39.8	0.01
Wheat flour	3.95	34.1	2.45

### Bio-distribution and pathophysiology

The T-2 toxin has widely been reported to be toxic to plants, mammals including humans, and in the dietary content for both vertebrates and invertebrates [82]. The magnitude of toxin injury is dependent on the administered dose and route of administration. It is readily absorbed, metabolized and nearly entirely excreted (80-90%) within 48 hours after ingestion and is uniformly distributed thoroughly the body without specific affinity for accumulation in any organ [62]. It has been observed in rodents that plasma concentrations attain peak levels after approx. 30 minutes. In one study, it was observed that upon radioactively tagging of the T-2 toxin, its half-life is less than 20 minutes in plasma. Furthermore, four hours after IV administration in pigs, 15-24% of the radioactivity was found in the GI tract and 4.7-5.2% in various other tissues such as the liver and muscles. The rapid onset of symptoms in minutes to hours supports a diagnosis of a chemical or toxin attack. Symptoms of T-2 toxicity are evidenced most frequently as reduced feed intake, weight loss, skin irritation, itching, diarrhea, bleeding, feed refusal, dyspnea, and vomiting [83,71] and range critically to haemorrhages and necrosis in the GI tract, reproductive organs and hematopoietic organs such as the bone marrow and spleen [61]. T-2 has also been reported to exert an important impact on reproductive performance in pigs [18]. Its toxicity varies according to its route of exposure, whereby it is highly toxic when ingested through the lungs as compared to other modes of ingestion [16]. Long-term effects range from mycotoxicosis (domestic animals), ATA (humans), inflammation of the GI mucosa, abdominal pain, vomiting, diarrhea, headache, generalized weakness, increased salivation, fatigue, and dizziness resulting in opportunistic secondary infections such as pneumonia.

### Effects on the immune system

T-2 toxin exposure results in leukopenia and cell depletion in lymphoid organs. It also inhibits erythropoiesis in the bone marrow and spleen and significantly impairs antibody production, reducing the proliferative response of lymphocytes and hindering the development of dendritic cells [61]. The T-2 toxin is also reported to inhibit IL-2 and IL-5 production by T cells. In addition to lymphocyte precursors, trichothecenes targets also include other hematopoietic progenitors, such as granulocyte, monocyte and erythrocyte colony-forming cells [72]. It has been observed that CD4/CD8 double-positive T cells from the thymus of young mice are highly sensitive to the T-2 toxin and that CD44^low^ and CD45^low^ cells, which are B lymphocyte precursors, are also highly sensitive to the T-2 toxin. Additionally, it has been documented to diminish immunoglobulin and cytokine levels. Studies have shown that prolonged low-dose exposure to this toxin can influence memory T cells and can have an adverse effect on the humoral response mediated by B lymphocytes and the secondary immune response in pigs [61]. It has also been established that the ingestion of low concentrations of the T-2 toxin alters TLR activation by decreasing the pattern recognition of pathogens, thus interfering with the initiation of inflammatory immune responses against bacteria and viruses [84]. The development of the immune-affinity 96-spot monolith array and high-sensitive chemiluminescent immunoassay investigation methods are highly promising means of detecting multiple mycotoxins in food samples [85, 86].

### Prophylaxis against the T-2 toxin: exploring herbal routes

The deleterious effects of exposure to the T-2 toxin can be minimized by means of detoxification remedies with natural substances. The consumption of a diet rich in probiotics, nutrients consisting of amino acids, enzymes, and lipids can aid in alleviating the symptoms of T-2 toxin damage. Several natural compounds, such as vitamins, provitamins, carotenoids, chlorophyll and its derivatives, phenolics, and selenium and synthetic compounds including butylated hydroxyanisole and butylated hydroxytoluene, have antioxidant properties that are believed to be efficacious against the T-2 toxin. The protective properties of antioxidants are most likely due to their superoxide anion scavenging ability, thereby protecting the cell membrane from mycotoxin induced injury [87]. It has been described that lycopene protects the liver against the T-2 toxin by reducing lipid peroxidation and modulating GSH metabolism *in vivo* [88]. It has also been reported that rutin can be used as an antioxidant in cases of T-2 toxicity in the liver of rats, as it aided in decreasing TBAR-induced lipid peroxidation, SOD, GST, total lipids and elevated total thiol and catalase levels as well as hemoglobin and hematocrit values [89]. It has been reported that lipid peroxides are formed *in vivo* by T-2 and that these effects can be partially counteracted by antioxidants such as vitamin E, though vitamin C is unable to exert the same protective effects [90]. Independent investigations have revealed that *Hippophae rhamnoides* (sea buckthorn) alone protected the immunosuppressant action of the T-2 toxin, but sea buckthorn and glucomannan in combination provided a synergistic effect with regard to protection against T-2 toxicity [91]. Researchers have indicated that owing to its natural healing potential, mucilage from quince seeds is a potential treatment of T-2-toxin-induced dermal injuries in rabbits [92].

If the toxin has been ingested orally, then super-activated charcoal can be utilized, as it adsorbs the ingested toxin and removes it from the GI tract, thus diminishing the ill effects of the toxin preventing it from causing cellular damage. Despite the fact that a variety of different strategies to combat mycotoxicosis have been established, the basis of the most encouraging methods consists of the addition of adsorbents to contaminated feed. The adsorbent material selectively binds toxins during digestion, preventing their absorption from the gastrointestinal tract and therefore decreasing their toxic effects. Researchers have demonstrated that a combination of modified glucomannan with organic selenium provides protection against the detrimental consequences of the consumption of T-2-toxin-contaminated feed resulting in toxin-induced antioxidant depletion and lipid peroxidation in the livers of chicken or hepatocytic cells [93, 94]. It has also been reported that small increases in the concentration of sodium selenite can confer highly significant protection against oxidative damage [95]. If natural remedies become ineffective, antifungal treatments may be prescribed on rare occasions. It has been reported that treating rats with Goji extract or charcoal can ameliorate the adverse effects of the T-2 toxin, but it was observed that Goji extract can be used as an antioxidant and antidote in place of charcoal against the T-2 toxin in mice [96]. However, several other complex mechanisms can also be utilized, which may involve modulation of metabolic detoxification pathways intercepting the action and formation of stable non-toxic complexes, and compounds having some degree of structural similarity between mycotoxin and protective agent molecules could aid in protection if assisted by competitive inhibition [87].

## REGULATORY GUIDELINES AND RECOMMENDATIONS

Due to the prevalence of the T-2 toxin in animal feed, several countries have formulated guidelines stipulating the maximal permissible limits of the toxin in products for animal use. China has limited the presence of the toxin in animal feed to 0.08 mg/kg, Iran and Canada have set the value for cattle animal feed to 0.1 mg/kg, while Canadian feed for poultry and swine has a maximal limit of 1.0 mg/kg of the T-2 toxin [97]. Currently, biochip array technology is in use for rapid multi-mycotoxin screening, including the screening of the T-2 and HT-2 toxins [98].

In the European Union, the maximum permitted content of T-2+HT-2 mycotoxins in feedstuffs (EC Directive 2002/32/EC, and EC Recommendations 2006/576/EC and 2013/165/EU) ranges from 0.1 ppm (mg/kg) for unprocessed wheat, rye and other cereals and 0.2 ppm for unprocessed barley (including malting barley) and maize up to 0.25 ppm for compound feed with the exception of feed for cats and 0.5 ppm for other cereal products designed for feed and compound feed. In addition, the established value is 2 ppm for oat milling products (husks) for feed and compound feed [99].

The maximum EU permitted content of T-2+HT-2 mycotoxins in human food (EC Regulation No 1881/2006 and EC Recommendation 2013/165/EU) ranges from 0.015 ppm for cereal-based foods for infants and young children; 0.025 ppm for bread and bakery wares, pastries, biscuits, cereal snacks or pasta; and 0.075 ppm for breakfast cereals including formed cereal flakes up to 0.05 ppm for cereal milling products and 0.1 ppm for cereal bran apart from oat bran, oat milling products other than oat bran, flaked oats and maize milling products, and 0.2 ppm for oat bran and flaked oats designed for direct human consumption [99]. However, no monitoring limits of T-2+ HT-2 coexist in North America, Latin America, or in the Asia/Oceania regions, including South Korea. T-2 and HT-2 toxin exposure levels in dietary contents were calculated using occurrence data retrieved from this study in 2009, mean body weights from the Korea Food and Drug Administration, and food consumption data from the Korean National Health and Nutrition Examination Survey (KNHANES 2008). Estimated daily exposure levels to T-2 and HT-2 toxins were calculated using the following formula:

$$\text{Estimated daily exposure}(\text{kg}/\text{bw}/\text{day})=\frac{\text{toxin concentration in samples}(\mu g/\text{kg})\times \text{consumption of six sample}(g/\text{day}/\text{person})}{\text{Mean body weight}(\text{kg}/\text{person})}$$

However, as compared to other mycotoxins, data pertaining to the occurrence of T-2 and HT-2 toxins in cereals and related products are very limited in South Korea [100, 81].

A number of strategies have been developed in an effort to inhibit the detrimental effects of the T-2 toxin. These include reducing the growth of mycotoxigenic fungi and reducing mycotoxin production, the detoxification of contaminated feed, and lowering the systemic availability level as soon as mycotoxins are ingested by an animal. Different types of radiation, i.e., γ-irradiation, X-rays, and ultraviolet light, have been explored for the decontamination of some mycotoxins, including the T-2 mycotoxin [101], and for their ability to control the growth of certain fungi [102,103], but these also have several disadvantages because radiation is effective only when applied to a thin layer of grain [104]. There are many energy inhibitors like sodium azide, 2-deoxy glucose, 2-4 dinitrophenol, ouabain which are well known to antagonize the uptake of T-2 toxin in murine lymphocytes. The inhibitors decreased the toxin level upto 40% by binding to High affinity site of T-2 toxin [105].

Additionally, post-harvest storage conditions also play a pivotal role in preventing mold growth and mycotoxin production. For instance, grains should be stored under conditions with moisture content levels of no more than 15% so as to avoid the formation of hotspots with high moisture, thus encouraging mold growth [51]. Hence, mycotoxins pose a grave public health hazard due to their deleterious side effects and the fact that they pose a severe threat to humans upon the consumption of residual traces in animal-derived food products originating from animals feeding on contaminated feedstuff [97].

### Potential hazardous agent

The T-2 toxin can act as a hazardous agent given that it can be absorbed via intact skin and cause blistering, irritation and systemic toxicity. The promptness of the toxic effect is evident by the fact that symptoms can begin to appear within seconds of exposure, though the demonstration of lethal effects requires a larger dosage of the T-2 toxin. It is a potentially critical biological warfare agent candidate, as the mode of ingestion of the T-2 toxin ranges diversely from food or water sources to various air-dispersal modes, including aerosols, droplets or smoke emanating from explosions. The LD_50_ of the T-2 toxin has been reported to be approximately 1 mg per kg of body weight. The T-2 toxin has been documented to have been used worldwide in various military conflicts during the period of 1975-81, and the aerosolized form has since widely become known as “yellow rain,” having been causally linked to thousands of casualties [97]. Primary symptoms of affected individuals immediately after exposure include skin blistering, a burning sensation, pain, pruritus, tenderness, and inflammation, and advanced symptoms in cases of lethal casualties include necrosis of the affected skin area accompanied by leathery blackening and sloughing off of exposed skin areas.

Interaction with the respiratory system initiates upon nasal contact and causes itching, pain, epistaxis, sneezing and rhinorrhea, which further advances the manifestation of pulmonary/tracheobronchial toxicity by exhibiting difficulty in breathing and coughing and wheezing. Oral and throat exposure to the toxin causes pain and blood-laden saliva and sputum. Ingestion of the toxin via the GI system leads to symptoms such as nausea, vomiting, anorexia, diarrhea associated with cramps and abdominal pain. If the toxin comes into contact with the eyes, it may result in redness and pain in the eyes, blurry vision, and a feeling of foreign body sensation. Following immediate effects, symptoms displaying systemic toxicity are manifested as generalized prostration, fatigue, weakness, dizziness, loss of coordination and ataxia. Fatal cases are the consequences of symptoms such as hypothermia, tachycardia, and hypotension resulting in death in a short span of time ranging from minutes to days [97].

### Isolation and decontamination

After T-2 toxin exposure, standard precautions should be taken according to set guidelines so as to minimize secondary exposure and damage. These consist of the removal of outer clothing and decontamination of exposed skin areas using soap and water. Eye contact with the toxin should be treated with profuse washing of the eyes with saline. Subsequently, isolation is non-essential after adequate exhaustive decontamination. Decontamination of the surrounding environments involves treatment with an alkaline hypochlorite solution (viz. 1% sodium hypochlorite and 0.1M NaOH) for a sufficient contact time interval. Porous substances with human exposure can be decontaminated only by meticulous UV light and an ozone exposure treatment. A RSDL (reactive skin decontamination lotion) kit is a topical decontamination solution that minimizes toxic effects from exposure to chemical warfare agents (VX and HD) and the T-2 toxin [106]. T-2 and other fabricated mycotoxins were assessed with immune-affinity monolithic arrays, which were proved as a sensitive, stable and economical tool to be used with food samples [85]. An alternative approach to lessen the degree of toxin exposure in feed is to decrease the level of bioavailability via the inclusion of mycotoxin detoxifying agents (mycotoxin detoxifiers) in the feed. These detoxifiers are mainly categorized into two different classes, i.e., mycotoxin binders (agents that adsorb the toxin in the gut, resulting in the excretion of the toxin-binder complex in feces) and mycotoxin modifiers or mycotoxin biotransforming agents (including microbes such as bacteria, fungi, yeast and enzymes that transform the toxin into non-toxic metabolites biologically) [51]. The adverse effects of the T-2 toxin are also reversed by the potential use of selenium and vitamin E on peripheral blood B lymphocytes [107].

### Methods for decontamination

#### Oxidation

Chemical methods for the oxidation of trichothecene toxins include a treatment with 0.25% NaOCl-0.025 mol/L NaOH for four hours. This has been shown to inhibit the biological activity of the T-2 toxin, and NaClO has also been acclaimed as a decontamination agent for the T-2 toxin and other trichothecenes [80].

Biological oxidation has proven to be more specific than chemical oxidation. It was observed that oxidation by means of hydroxylation in animal bodies resulted in the addition of a hydroxyl group at the C-3′ position of the C-8 substituent of type A trichothecenes. Various animal species such as mice, rats, monkeys, rabbits, chicken, swine, cows and even the shrub *Baccharis* spp. have exhibited oxidation of the T-2 toxin via hydroxylation to the 3′-hydroxy T-2 toxin (3′-OH T-2) and/or the 3′-hydroxy HT-2 toxin (3′-OH HT-2). It was observed that the formation of 3′-OH T-2 and 3′-OH HT-2 toxins took place in microsomes in the presence of NADPH [80].

#### Conjugation by glycosidation

Trichothecene mycotoxins can be conjugated by glycosidation to yield glucuronides and glucosides. The most important biochemical pathway is glucuronidation for the metabolism of the T-2 toxin and the HT-2 toxin in animal and human systems. The glucuronidation of trichothecenes has reported to be achieved by microsomal glucuronyl transferase or glucuronidase from the rat liver [108].

#### Bentonite

Studies have reported that bentonite feeding (5-10%) inhibits the toxic effects of T-2 by decreasing intestinal absorption and increasing fecal excretion of the toxin [109]. Bentonite has shown the potential to bind aflatoxin, sterigmatocystin, zearalenone and the T-2 toxin [110].

### Future perspectives

The identification of detoxification agents against T-2 mycotoxicosis is more important, and its side effects need to be assessed. Future potential applications of the detoxification of mycotoxin by microorganisms, enzymes and genes could prove to be beneficial. These detoxification enzymes should have pronounced capabilities to eliminate mycotoxins from human and animal systems and from foods and feedstocks. Beneficial detoxification genes used could be cloned and expressed in microorganisms to develop recombinant microorganisms that are biologically and ethically appropriate for industrial-scale enzyme production and purification. The development of host resistance strategies and characterization studies can also be employed to realize efficient methods which target T-2 toxin decontamination. RNAi gene silencing and gene mapping can prove to play a pivotal role in building strategies to increase the contribution of selected resistance-associated proteins in seed/crop resistance to T-2 contamination [111].

#### Plausible therapeutic uses

T-2 mycotoxin and their derivatives have been attributed a diverse range of abilities, including their application as growth promoters, antibiotics, and a range of other drugs. The trichothecene family has been credited with numerous biological properties consisting of antiviral abilities (chiefly as Herpes replication inhibitors), immunotoxic activities, antileukemic and antimalarial capabilities [23]. This vast amount of the aggregated understanding of mycotoxins has opened a new era of applications utilizing an amalgamation of toxin dexterity factors with progress made with scientific techniques in various fields such as immunology, biotechnology, molecular biology, cell biology and nanotechnology in order to develop target-specific strategies that can adapt a fatal toxin into a potential therapeutic agent.

**Figure 7:**
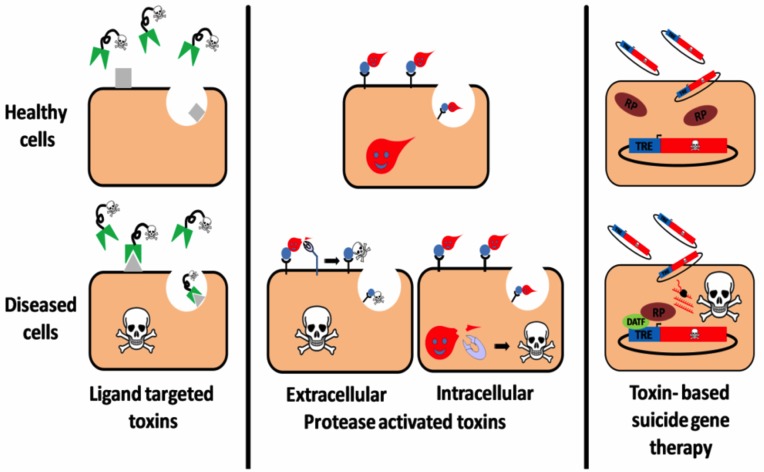
Three targeting strategies in a T-2-toxin-based therapy (Reproduced from Shapira A 2010 with the permission of the Toxin Journal).

The major strategies developed in this area include toxins which target ligands being administered, which upon internalization, target and attack diseased cells while specifically sparing unexposed cells that do not display receptors on their surfaces. Another approach follows a protease-activated toxin strategy whereby the toxin is cleaved via biotechnological engineering and activated upon interaction with a disease-related intracellular/extracellular protease. This cleavage is hypothesized to enhance cell binding, which may lead to a signal transduction cascade, translocation, stabilization or increased catalytic activity of the toxin moiety in targeted cells, resulting in their suppression. Amongst potential therapeutic usage strategies, toxin-based suicide gene therapies are also promising. These consist of a toxin polypeptide-encoding DNA construct being delivered to an assorted cell population (Figure 7). A specific transcription regulator oversees the regulation of the DNA construct expression [112, 23]. Thus, advancements of such strategies could prove to be beneficial and provide us with a tangible solution to the ever-increasing medical challenges and applications related to these toxins.

## CONCLUSIONS

Fungal secondary metabolites such as the T-2 toxin have had severe adverse effects and continue to poison farm animals worldwide. The T-2 toxin and its metabolites exist in various countries but are mainly found in tropical and subtropical regions, such as South Korea. These toxins contaminate animal feedstuff and their production is aided by the grain moisture content and weather conditions in the affected areas. The symptoms of toxicity are diverse, affecting the GI tract mucosa and digestion process and causing skin blistering, edema, irritation, necrosis and apoptosis. These all present bio-related threats to humans and can induce oxidative stress, causing DNA damage, inhibiting protein synthesis, and damaging lipids.

These lethal properties of the toxin support its candidature as a fatal biological warfare agent. Despite various guidelines and regulations established regarding its usage and detection strategies pertaining to maximum permissible limits in feed and food stocks, its presence can prove to be toxic. Presently, T-2 toxin treatments of induced damage emphasize mainly the use of natural substances, probiotics, and amino acids, and the quest for a precise antidote against the toxin continues to date. Therefore, stringent regulations must be established and quarantine activities need to be undertaken in order to prevent planned/unplanned exposure on a large scale.

## Abbreviations

T-2: Type of trichothecene toxin
HT-2: hydroxy trichothecene-2
LD50: lethal dose to kill 50 percent of test sample
MAPK: mitogen activated protein kinase
ERK ½: extracellular signal-regulated kinase ½
ATF: activating transcription factor
JNK: c-Jun NH_2_-terminal kinases
